# Prognostic value of hemoglobin-to-serum creatinine ratio in patients with coronary artery disease and reduced kidney function following percutaneous coronary intervention

**DOI:** 10.3389/fcvm.2026.1868952

**Published:** 2026-09-10

**Authors:** Sini Fang, Huanting Liu, Youcheng Wang, Guangjie Wang, Yumeng Lei, Alian Wei, Xiyuan Mo, Liqiu Yan

**Affiliations:** 1 Department of Cardiology, The Affiliated Dongguan Songshan Lake Central Hospital, Guangdong Medical University, Dongguan, Guangdong, China; 2 Dongguan Key Laboratory of Cardiovascular Aging and Myocardial Regeneration, Dongguan Cardiovascular Institute, Dongguan, China; 3 Department of Cardiology, Cangzhou Central Hospital Affiliated to Hebei Medical University, Cangzhou, Hebei, China; 4 School of Public Health, Guangdong Medical University, Guangdong, China

**Keywords:** cardiovascular disease, hemoglobin-to-serum creatinine ratio, percutaneous coronary intervention, prognosis, reduced kidney function

## Abstract

**Objective:**

This investigation sought to assess the prognostic significance of the hemoglobin-to-serum creatinine (Hb/SCr) ratio for long-term survival among patients with coexisting coronary artery disease (CAD) and reduced kidney function after percutaneous coronary intervention (PCI).

**Methods:**

This retrospective study encompassed 2,310 patients who underwent PCI and had concomitant CAD and reduced kidney function. The cohort median Hb/SCr value of 1.60 was used as a pragmatic reference threshold to classify patients into low (<1.60) and high (≥1.60) Hb/SCr groups. The predictive significance of the Hb/SCr ratio regarding cardiac-cause mortality (CCM) and all-cause mortality (ACM) over a follow-up period of up to 60 months was evaluated employing Kaplan–Meier analysis and multivariable Cox proportional hazards models.

**Results:**

During 60 months of follow-up, 56 cardiac-cause deaths (2.4%) and 87 all-cause deaths (3.8%) occurred. Kaplan–Meier analysis showed significantly higher cumulative mortality in the low Hb/SCr group for both CCM (log-rank *P* = 0.002263) and ACM (log-rank *P* = 0.002349). The Hb/SCr ratio showed modest discrimination for CCM (AUC 0.663, 95% CI 0.585–0.742) and ACM (AUC 0.614, 95% CI 0.548–0.680). Compared with Hb/SCr < 1.60, Hb/SCr ≥ 1.60 was associated with lower adjusted risks of CCM (HR 0.49, 95% CI 0.27–0.90; *P* = 0.021) and ACM (HR 0.59, 95% CI 0.37–0.94; *P* = 0.028). Similar associations were observed when the ratio was analysed as a continuous variable (CCM: HR 0.28, 95% CI 0.14–0.57; ACM: HR 0.42, 95% CI 0.23–0.77).

**Conclusion:**

A lower Hb/SCr ratio independently predicts increased long-term risks of CCM and ACM in patients with CAD and reduced kidney function after PCI. Given its modest discriminative ability, this readily available ratio may serve as a complementary marker for risk stratification rather than a standalone predictive tool, potentially helping to identify patients who warrant closer post-procedural surveillance.

## Introduction

Cardiovascular disease (CVD) continues to represent a primary contributor to global morbidity and mortality, responsible for roughly 20 million deaths each year and imposing substantial strain on healthcare systems worldwide (1, 2). Coronary artery disease (CAD) represents the most prevalent type of CVD and remains a major contributor to cardiovascular mortality (1). Percutaneous coronary intervention (PCI) has become a central therapeutic strategy for CAD and is widely performed to reestablish coronary perfusion, relieve ischemic manifestations, and improve short-term clinical outcomes. Despite continuing progress in interventional techniques and pharmacologic treatment, a substantial proportion of patients undergoing PCI still experience unfavorable long-term outcomes, including recurrent cardiovascular events and death (3, 4). Therefore, the identification of reliable and readily accessible prognostic biomarkers for risk stratification remains a major issue in clinical practice.

Among the numerous factors affecting the prognosis of patients with CAD, anemia and reduced kidney function have long been recognized as important predictors of adverse cardiovascular outcomes (5–9). Anemia compromises oxygen delivery and aggravates myocardial ischemia, thereby increasing cardiac workload and contributing to myocardial injury. In contrast, reduced kidney function is linked to chronic inflammation, oxidative stress, endothelial dysfunction, and accelerated atherosclerosis. Notably, anemia and reduced kidney function frequently coexist in patients with CAD, particularly in those undergoing PCI (9, 10). The interplay between these two conditions may further aggravate cardiovascular injury through multiple pathophysiological mechanisms, thereby increasing the risks of cardiovascular events and mortality.

As a composite indicator, the hemoglobin-to-serum creatinine (Hb/SCr) ratio has recently attracted clinical interest because it integrates the evaluation of anemia and renal impairment into a single parameter (11–13). Compared with isolated laboratory measures, this ratio may more effectively reflect the balance between oxygen-carrying capacity and reduced kidney function, both of which are closely linked to cardiovascular outcomes. Previous studies have linked the Hb/SCr ratio with elevated adverse outcome risk in CAD patients (11). However, existing research has predominantly concentrated on the general CAD population or patients with acute coronary syndrome, and the prognostic significance of the Hb/SCr ratio in CAD patients with reduced kidney function undergoing PCI remains inadequately established. Reduced kidney function markedly increases the risks of cardiovascular complications and mortality after PCI. This condition is often accompanied by anemia and metabolic disorders, which may further aggravate adverse cardiovascular outcomes (14–16). Therefore, the identification of simple, cost-effective, and readily accessible biomarkers to improve risk prediction in this vulnerable population is of substantial clinical significance.

The primary objective of this investigation was to establish the link between the Hb/SCr ratio and long-term survival among CAD patients with concurrent reduced kidney function following PCI. Additionally, the study evaluated whether this index could reliably stratify risk and predict both cardiac-cause mortality (CCM) and all-cause mortality (ACM) during extended follow-up.

## Methods

### Study population

**Figure 1:**
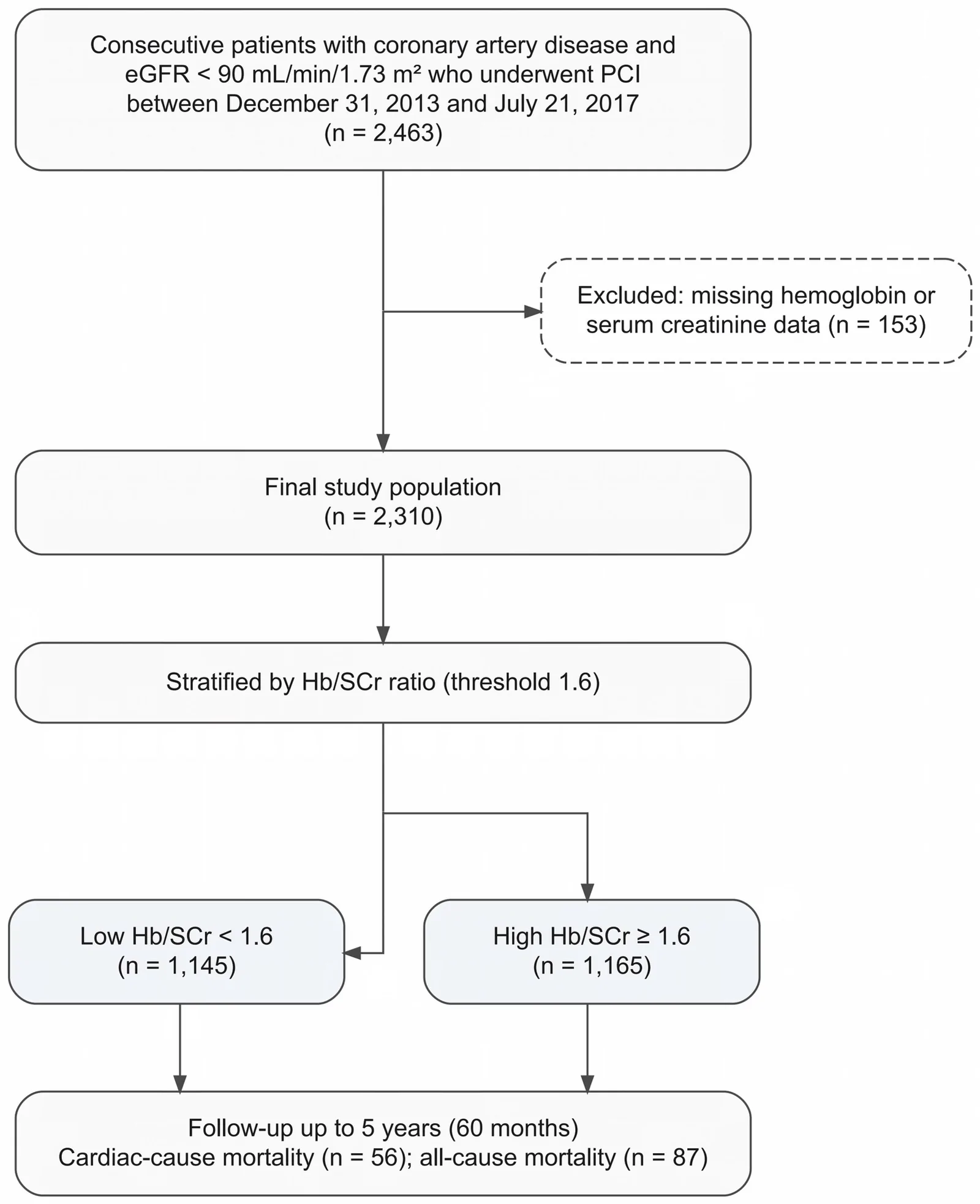
Study flowchart. Flow diagram detailing the selection process of patients with coronary artery disease and reduced kidney function who underwent percutaneous coronary intervention (PCI).

This retrospective cohort analysis was conducted at Cangzhou Central Hospital between December 31, 2013 and July 21, 2017. The study population comprised patients with CAD who underwent PCI during this period. Institutional review board approval was secured from Cangzhou Central Hospital, an affiliated hospital of Hebei Medical University (Approval No.: 2023-144-02), and written informed consent was procured from all participants. The cohort comprised adults aged 18 years or older with a confirmed CAD diagnosis who had received PCI. Additionally, eligible patients were required to have concurrent reduced kidney function, characterized as an eGFR < 90 mL/min/1.73 m^2^. This threshold was selected to include patients across a broad spectrum of reduced kidney function, from mildly to severely decreased GFR, rather than restricting the cohort to patients with eGFR < 60 mL/min/1.73 m^2^. Previous studies have shown that even mildly decreased eGFR is independently associated with adverse cardiovascular outcomes following PCI (17). We note that KDIGO G2 alone, in the absence of markers of kidney damage such as albuminuria, does not meet the KDIGO diagnostic criteria for chronic kidney disease (CKD). Accordingly, the present study used eGFR-based stratification to characterize kidney function rather than to establish a diagnosis of CKD. Patients undergoing dialysis, individuals with end-stage renal disease, and those diagnosed with active malignancy were excluded. In total, 2,463 patients underwent initial screening. Patients with missing hemoglobin or serum creatinine values were excluded during screening (*n* = 153), because the Hb/SCr ratio could not be calculated. Missing values in other baseline variables, each with less than 10% missingness, were handled using multiple imputation in R, with 10 imputed datasets generated. Variables included in the final Cox regression models had no missing values; therefore, all 2,310 patients contributed to these analyses (Figure 1).

### Data collection and definitions

Baseline demographic, clinical, laboratory, echocardiographic, and angiographic data were obtained from the electronic medical records. Variables included age, sex, smoking status, hypertension, diabetes mellitus, chronic obstructive pulmonary disease, atrial fibrillation, previous myocardial infarction, previous stroke, previous PCI, and clinical presentation (stable angina, unstable angina, STEMI, or NSTEMI). Laboratory and cardiac function variables included hemoglobin, white blood cell count, platelet count, total cholesterol, blood glucose, urea, serum creatinine, and left ventricular ejection fraction. Kidney function was assessed using eGFR and categorized as KDIGO G2 (60–89 mL/min/1.73 m^2^), G3 (30–59 mL/min/1.73 m^2^), or G4–G5 (<30 mL/min/1.73 m^2^). The number of diseased coronary vessels was obtained from angiographic reports and electronic imaging systems.

Before PCI, baseline venous blood samples were procured from all hospitalized patients. Hemoglobin (Hb, g/L) and serum creatinine (SCr, μmol/L) levels were determined using standard laboratory protocols. The Hb/SCr ratio was calculated as hemoglobin (g/L) divided by serum creatinine (μmol/L). Additionally, eGFR was ascertained through the abbreviated Modification of Diet in Renal Disease equation.

### Outcomes and follow-up

The main endpoints were CCM and ACM, up to 60-month follow-up. Follow-up data were obtained by trained healthcare professionals who were blinded to baseline Hb/SCr ratio levels through structured telephone interviews or outpatient visits. Follow-up duration represented the time interval between the PCI date and either endpoint event occurrence or completion of the 60-month observation period.

### Statistical analysis

Statistical analyses were performed employing R software (version 4.4.2). Restricted cubic spline models with four knots were used to assess the dose–response relationship between the Hb/SCr ratio and mortality and to evaluate potential nonlinearity, with adjustment for age, sex, smoking, diabetes, hypertension, history of stroke, and left ventricular ejection fraction. Because no evidence of nonlinearity or a distinct inflection point was observed, the cohort median Hb/SCr value of 1.60 was used as a pragmatic reference threshold for subsequent categorical analyses. Patients were accordingly stratified into low (<1.6) and high (≥1.6) Hb/SCr groups. ROC analysis was performed separately to evaluate discriminative ability. The performance of the Hb/SCr ratio was compared with that of hemoglobin and serum creatinine alone using DeLong's test, and sensitivity, specificity, and positive and negative predictive values at the selected threshold were calculated. The Kolmogorov–Smirnov test assessed continuous variable distribution. Based on data distribution, continuous variables were denoted as mean ± standard deviation (SD) or median with interquartile range (IQR) and were examined employing the Student's *t*-test or Mann–Whitney *U* test, as appropriate. Categorical variables were denoted as frequencies and percentages, and between-group differences were evaluated utilizing the Chi-square test. Kaplan–Meier curves were constructed to compare survival between the low and high Hb/SCr groups, with between-group differences assessed using the log-rank test. In addition, quartile-based analyses with tests for linear trend were performed to further evaluate the consistency of the association between the Hb/SCr ratio and clinical outcomes.

Age and sex were retained as basic demographic covariates, while additional covariates were selected based on univariable Cox analysis (*P* < 0.05). Three progressively adjusted Cox proportional hazards models were then constructed to evaluate the independent prognostic value of the Hb/SCr ratio. Model 1 was unadjusted; Model 2 was adjusted for age and sex; and Model 3 was additionally adjusted for smoking, diabetes, hypertension, history of stroke, and left ventricular ejection fraction. The results are denoted as hazard ratio (HR) and 95% confidence interval (CI). Subgroup analyses were performed according to age (<60 vs. ≥60 years), sex, hypertension, chronic obstructive pulmonary disease, diabetes, number of stents (1 vs. ≥2), myocardial infarction status (MI vs. non-MI), and eGFR category (G2 vs. G3). Cox proportional hazards models were fitted within each subgroup to estimate HRs and 95% CIs for cardiac-cause mortality, and interaction effects were assessed using likelihood ratio tests. The G4–G5 category was not displayed because sparse data precluded reliable estimation.

The proportional hazards assumption was assessed using scaled Schoenfeld residuals; the stability of the AUC estimates was assessed using 1,000 bootstrap resamples; and the 5-year incremental predictive value of the Hb/SCr ratio was evaluated using integrated discrimination improvement and continuous net reclassification improvement with 500 perturbation resamples.

## Results

The baseline demographic and clinical characteristics for the study population are presented in Table 1. Among the 2,310 patients, the mean age was 64.97 ± 8.79 years, and 58.3% were male. Patients in the low Hb/SCr ratio group demonstrated advanced age and exhibited elevated proportions of males, smokers, and individuals with diabetes mellitus relative to those in the high-ratio group. The low Hb/SCr group additionally exhibited a more severe baseline clinical status. Kidney function was markedly poorer in this group, as reflected by higher urea levels and a greater proportion of patients with eGFR categories G3 or G4–G5. Patients in the low Hb/SCr group had higher proportions of STEMI and NSTEMI, whereas stable and unstable angina were more common in the high Hb/SCr group. During follow-up, unadjusted analysis revealed that both CCM (3.4% vs. 1.5%, *P* = 0.004) and ACM (5.0% vs. 2.6%, *P* = 0.003) were markedly elevated in the low Hb/SCr ratio group.

**Table 1:** Baseline demographic and clinical characteristics and clinical outcomes of the study population stratified by the Hb/SCr ratio.

Characteristic	Total (*N* = 2310)	Hb/SCr ratio low (*N* = 1145)	Hb/SCr ratio high (*N* = 1165)	*P* value
Age, years	64.97 ± 8.79	65.49 ± 9.31	64.47 ± 8.22	0.005
Sex				<0.001
Males	1,347 (58.3%)	831 (72.6%)	516 (44.3%)	
Females	963 (41.7%)	314 (27.4%)	649 (55.7%)	
Hemoglobin, g/L	132.00 (121, 143)	128 (115, 138)	137.00 (127, 147)	<0.001
WBC, ×10⁹/L	7.08 (5.80, 8.89)	7.32 (5.92, 9.44)	6.84 (5.71,8.53)	<0.001
Total cholesterol, mmol/L	4.46 ± 1.00	4.34 ± 0.98	4.59 ± 1.02	<0.001
Platelet count, ×10⁹/L	218.16 ± 59.71	212.96 ± 63.27	223.27 ± 55.55	<0.001
Blood glucose, mmol/L	6.50 (5.40, 8.70)	6.60 (5.40, 9.03)	6.40 (5.38, 8.37)	0.028
Serum creatinine, μmol/L	84.00 (72.00, 94.00)	93.00 (85.00,104.00)	74.00 (66.00,84.00)	<0.001
LVEF, %	60.00 (60.00, 62.00)	60.00 (60.00, 63.00)	60.00 (60.00,61.00)	<0.001
Urea, mmol/L	6.10 (5.00, 7.50)	6.80 (5.50, 8.30)	5.60 (4.70, 6.70)	<0.001
eGFR categories				<0.001
G2	2,042 (88.4%)	878 (76.7%)	1,164 (99.9%)	
G3	246 (10.6%)	245 (21.4%)	1 (0.1%)	
G4–G5	22 (1.0%)	22 (1.9%)	0 (0.0%)	
Clinical Diagnosis				<0.001
Stable angina pectoris	1,064 (46.1%)	462 (40.3%)	602 (51.7%)	
Unstable angina pectoris	539 (23.3%)	242 (21.1%)	297 (25.5%)	
STEMI	51 (2.2%)	36 (3.1%)	15 (1.3%)	
NSTEMI	656 (28.4%)	405 (35.4%)	251 (21.5%)	
COPD	37 (1.6%)	22 (1.9%)	15 (1.3%)	0.295
Atrial fibrillation	89 (3.9%)	48 (4.2%)	41 (3.5%)	0.464
Diabetes mellitus	525 (22.7%)	287 (25.1%)	238 (20.4%)	0.009
Previous stroke	252 (10.9%)	149 (13.0%)	103 (8.8%)	0.002
Smoking	261 (11.3%)	149 (13.0%)	112 (9.6%)	0.012
Previous PCI	302 (13.1%)	160 (14.0%)	142 (12.2%)	0.226
Previous MI				0.122
No	2,114 (91.5%)	1,037 (90.6%)	1,077 (92.4%)	
Yes	196 (8.5%)	108 (9.4%)	88 (7.6%)	
Hypertension	1,551 (67.1%)	767 (67.0%)	784 (67.3%)	0.909
Number of diseased vessels				0.167
1	506 (21.9%)	232 (20.3%)	274 (23.5%)	
2	793 (34.3%)	401 (35.0%)	392 (33.6%)	
3	1,011 (43.8%)	512 (44.7%)	499 (42.8%)	
Cardiac-cause mortality	56 (2.4%)	39 (3.4%)	17 (1.5%)	0.004
All-cause mortality	87 (3.8%)	57 (5.0%)	30 (2.6%)	0.003

WBC, white blood cell; LVEF, left ventricular ejection fraction; eGFR, estimated glomerular filtration rate; STEMI, ST-segment elevation myocardial infarction; NSTEMI, non-ST-segment elevation myocardial infarction; COPD, chronic obstructive pulmonary disease; MI, myocardial infarction; PCI, percutaneous coronary intervention.

eGFR categories: G2 (60–89), G3 (30–59), G4–G5 (<30 mL/min/1.73 m^2^), per KDIGO classification.

**Figure 2:**
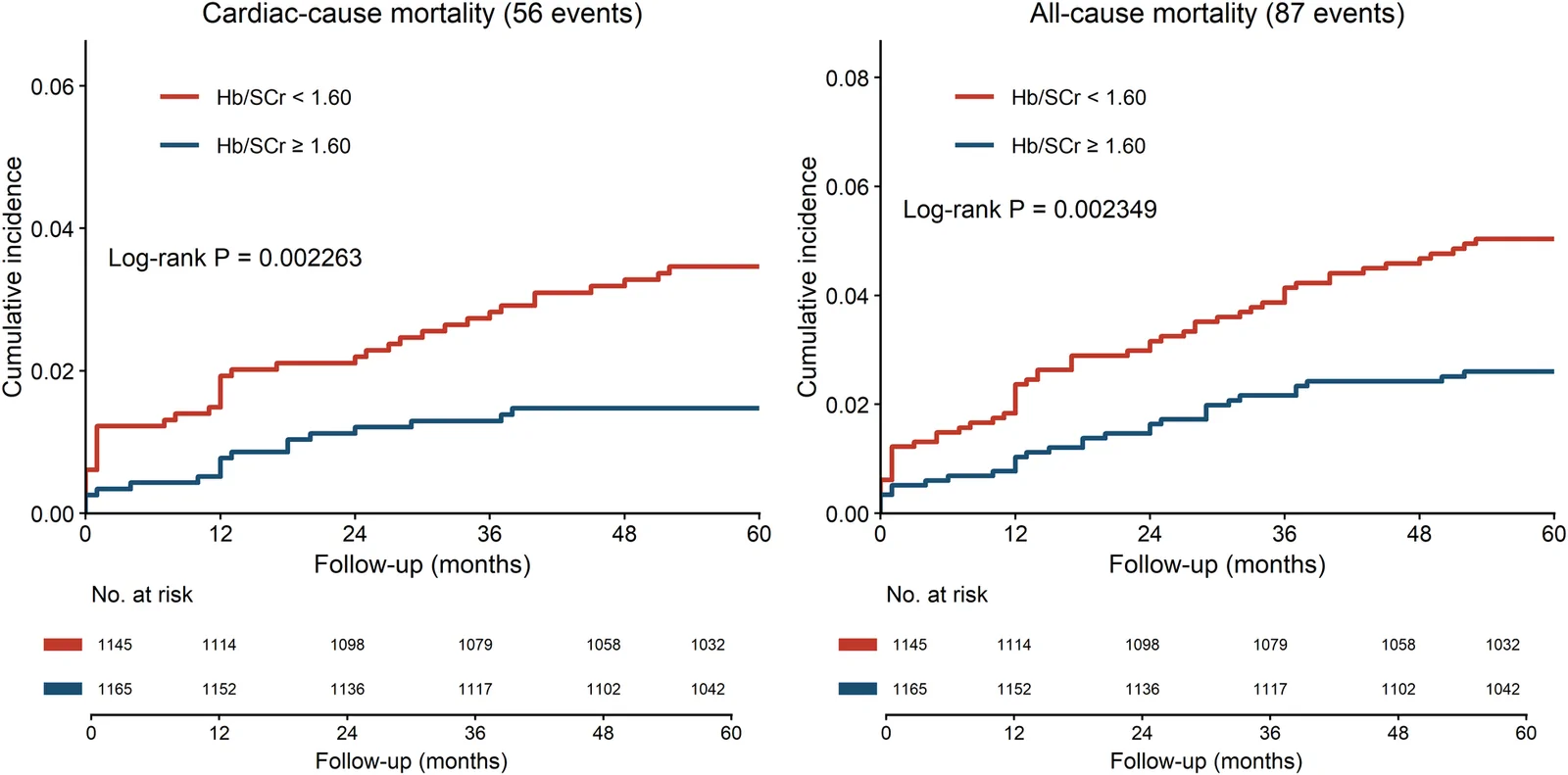
Kaplan–Meier analysis for cardiac-cause mortality (left; 56 events) and all-cause mortality (right; 87 events), stratified by the Hb/SCr ratio. Differences were assessed using the log-rank test.

Kaplan–Meier analysis demonstrated that patients in the low Hb/SCr group had higher cumulative risks of both CCM and ACM than those in the high Hb/SCr group (CCM: log-rank *P* = 0.002263; ACM: log-rank *P* = 0.002349; Figure 2). In the univariate Cox models (Tables 2, 3), the Hb/SCr ratio showed a strong inverse association with both mortality outcomes. Each 1-unit increase in the continuous Hb/SCr ratio was associated with a 79% lower risk of CCM (HR = 0.21, 95% CI 0.11–0.41, *P* < 0.001) and a 68% lower risk of ACM (HR = 0.32, 95% CI 0.18–0.57, *P* < 0.001). Similar findings were observed in the categorical analysis, in which patients with an Hb/SCr ratio ≥1.6 had significantly lower risks of CCM (HR = 0.42, 95% CI 0.24–0.75, *P* = 0.003) and ACM (HR = 0.51, 95% CI 0.33–0.79, *P* = 0.003). These associations remained significant after multivariable adjustment. In the fully adjusted Model 3, each 1-unit increase in the Hb/SCr ratio was independently associated with lower risks of CCM (HR = 0.28, 95% CI 0.14–0.57, *P* < 0.001) and ACM (HR = 0.42, 95% CI 0.23–0.77, *P* = 0.005). Likewise, an Hb/SCr ratio ≥1.6 remained independently associated with lower risks of CCM (HR = 0.49, 95% CI 0.27–0.90, *P* = 0.021) and ACM (HR = 0.59, 95% CI 0.37–0.94, *P* = 0.028). The global tests showed no evidence of violation of the proportional hazards assumption in any Cox model, and the assumption was satisfied for the Hb/SCr term in all models (global *P* = 0.556 and 0.307 for CCM; *P* = 0.462 and 0.233 for ACM; Supplementary Table S3). Given the limited number of outcome events, several confidence intervals were relatively wide and should be interpreted cautiously.

**Table 2:** Cox proportional hazards analysis for cardiac-cause mortality.

Variable	Model 1 HR (95% CI)	*P* value	Model 2 HR (95% CI)	*P* value	Model 3 HR (95% CI)	*P* value
Hb/SCr ratio (categorical)						
Group 1 (<1.6)	Reference		Reference		Reference	
Group 2 (≥1.6)	0.42 (0.24−0.75)	0.003	0.45 (0.25–0.83)	0.010	0.49 (0.27–0.90)	0.021
Hb/SCr ratio (continuous)	0.21 (0.11–0.41)	<0.001	0.25 (0.13–0.50)	<0.001	0.28 (0.14–0.57)	<0.001

Model 1: unadjusted. Model 2: adjusted for age and sex. Model 3: adjusted for age, sex, smoking, diabetes, hypertension, history of stroke, and left ventricular ejection fraction. The continuous analysis expresses risk per 1-unit increase in the Hb/SCr ratio.

Hb/SCr, hemoglobin-to-serum creatinine ratio; HR, hazard ratio; CI, confidence interval.

**Table 3:** Cox proportional hazards analysis for all-cause mortality.

Variable	Model 1 HR (95% CI)	*P* value	Model 2 HR (95% CI)	*P* value	Model 3 HR (95% CI)	*P* value
Hb/SCr ratio (categorical)						
Group 1 (<1.6)	Reference		Reference		Reference	
Group 2 (≥1.6)	0.51 (0.33–0.79)	0.003	0.58 (0.36–0.93)	0.016	0.59 (0.37–0.94)	0.028
Hb/SCr ratio (continuous)	0.32 (0.18–0.57)	<0.001	0.42 (0.23–0.74)	0.003	0.42 (0.23–0.77)	0.005

Model 1: unadjusted. Model 2: adjusted for age and sex. Model 3: adjusted for age, sex, smoking, diabetes, hypertension, history of stroke, and left ventricular ejection fraction. The continuous analysis expresses risk per 1-unit increase in the Hb/SCr ratio.

Hb/SCr, hemoglobin-to-serum creatinine ratio; HR, hazard ratio; CI, confidence interval.

**Figure 3:**
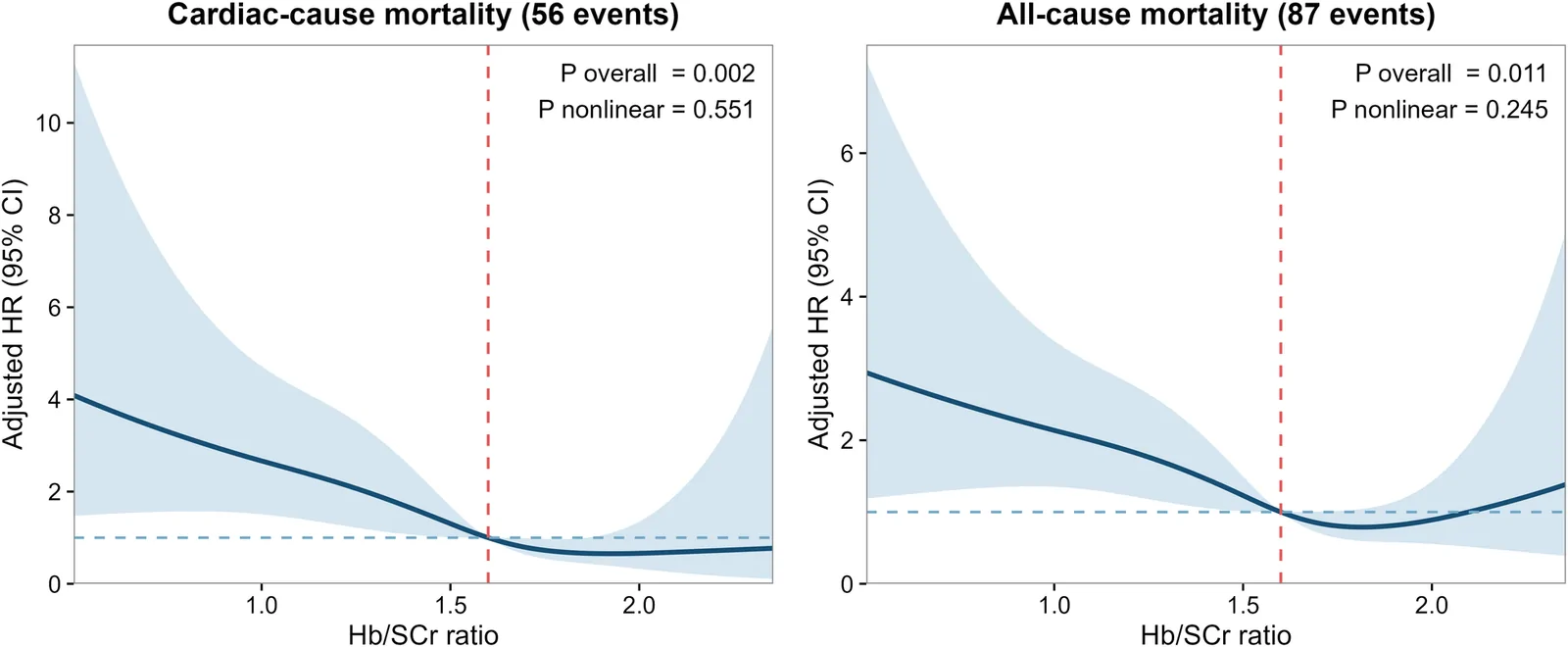
Restricted cubic spline analyses of the associations between the Hb/SCr ratio and cardiac-cause mortality (left) and all-cause mortality (right). Solid lines represent adjusted hazard ratios, and shaded areas indicate 95% confidence intervals. Models were adjusted for age, sex, smoking, diabetes, hypertension, history of stroke, and left ventricular ejection fraction. The vertical red dashed line indicates the cohort median Hb/SCr value of 1.60 used as the reference threshold for categorical analyses.

Restricted cubic spline analyses adjusted for age, sex, smoking, diabetes, hypertension, history of stroke, and left ventricular ejection fraction demonstrated significant inverse associations between the Hb/SCr ratio and both cardiac-cause mortality (*P* for overall = 0.002) and all-cause mortality (*P* for overall = 0.011), with no evidence of nonlinearity (*P* for nonlinearity = 0.551 and 0.245, respectively) (Figure 3).

**Figure 4:**
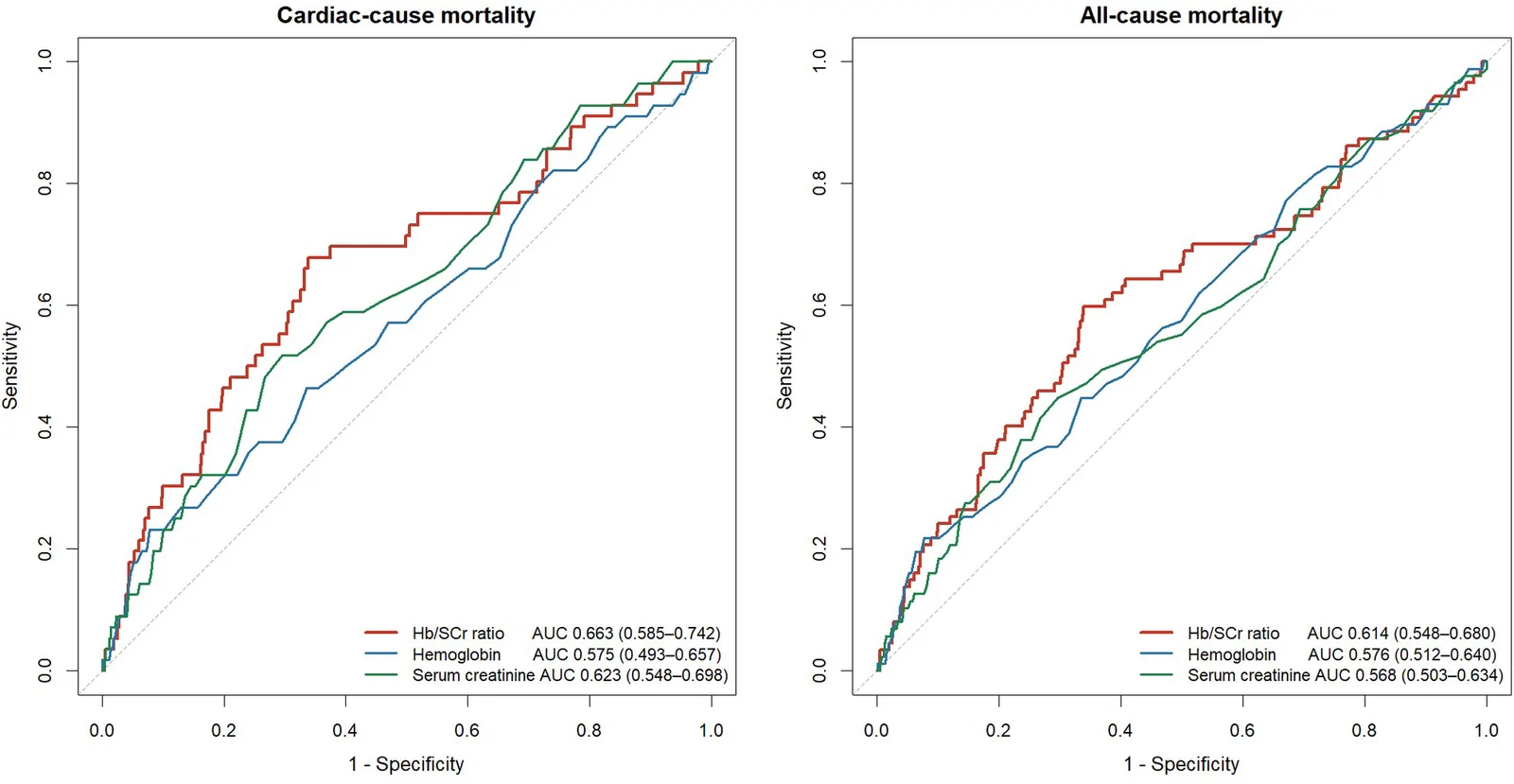
Receiver operating characteristic curves comparing the Hb/SCr ratio with hemoglobin and serum creatinine alone for cardiac-cause mortality (left) and all-cause mortality (right).

To assess the discriminatory capacity of the Hb/SCr ratio in predicting long-term survival, a ROC curve was generated. The analysis produced an area under the curve of 0.663 (95% CI 0.585–0.742) for CCM and 0.614 (95% CI 0.548–0.680) for ACM, as shown in Figure 4. At the selected threshold of 1.6, sensitivity and specificity were 69.6% and 50.9% for CCM, and 65.5% and 51.1% for ACM, respectively. The corresponding positive and negative predictive values were 3.4% and 98.5% for CCM, and 5.0% and 97.4% for ACM, reflecting the low event rates in this cohort. Compared with its individual components, the Hb/SCr ratio showed better discrimination for CCM than hemoglobin alone (AUC: 0.663 vs. 0.575, *P* = 0.024) and for ACM than serum creatinine alone (AUC: 0.614 vs. 0.568, *P* = 0.037). The remaining pairwise comparisons were not statistically significant (Supplementary Table S1). Bootstrap resampling with 1,000 replicates yielded confidence intervals similar to the analytically derived intervals (95% CI 0.582–0.741 for CCM and 0.549–0.682 for ACM) (Supplementary Table S2).

Quartile-based analyses further demonstrated a graded inverse association between the Hb/SCr ratio and mortality. Compared with the lowest quartile, the adjusted HRs for CCM decreased progressively across increasing quartiles (Q2: 0.46, 95% CI 0.23–0.93; Q3: 0.40, 95% CI 0.19–0.86; Q4: 0.34, 95% CI 0.15–0.79), with a significant linear trend (*P* for trend = 0.004). For ACM, quartile estimates were not strictly monotonic, although the trend test was statistically significant (*P* for trend = 0.026; Supplementary Table S4).

Adding the Hb/SCr ratio to the clinical model resulted in modest but statistically significant improvements in discrimination, as reflected by the integrated discrimination improvement (IDI = 0.007, 95% CI 0.0001–0.0289, *P* = 0.048 for CCM; IDI = 0.005, 95% CI 0.0001–0.0217, *P* = 0.032 for ACM). However, continuous net reclassification improvement was not statistically significant for either endpoint (0.141, 95% CI −0.031 to 0.279, *P* = 0.100 for CCM; 0.085, 95% CI −0.073 to 0.187, *P* = 0.299 for ACM; Supplementary Table S5).

**Figure 5:**
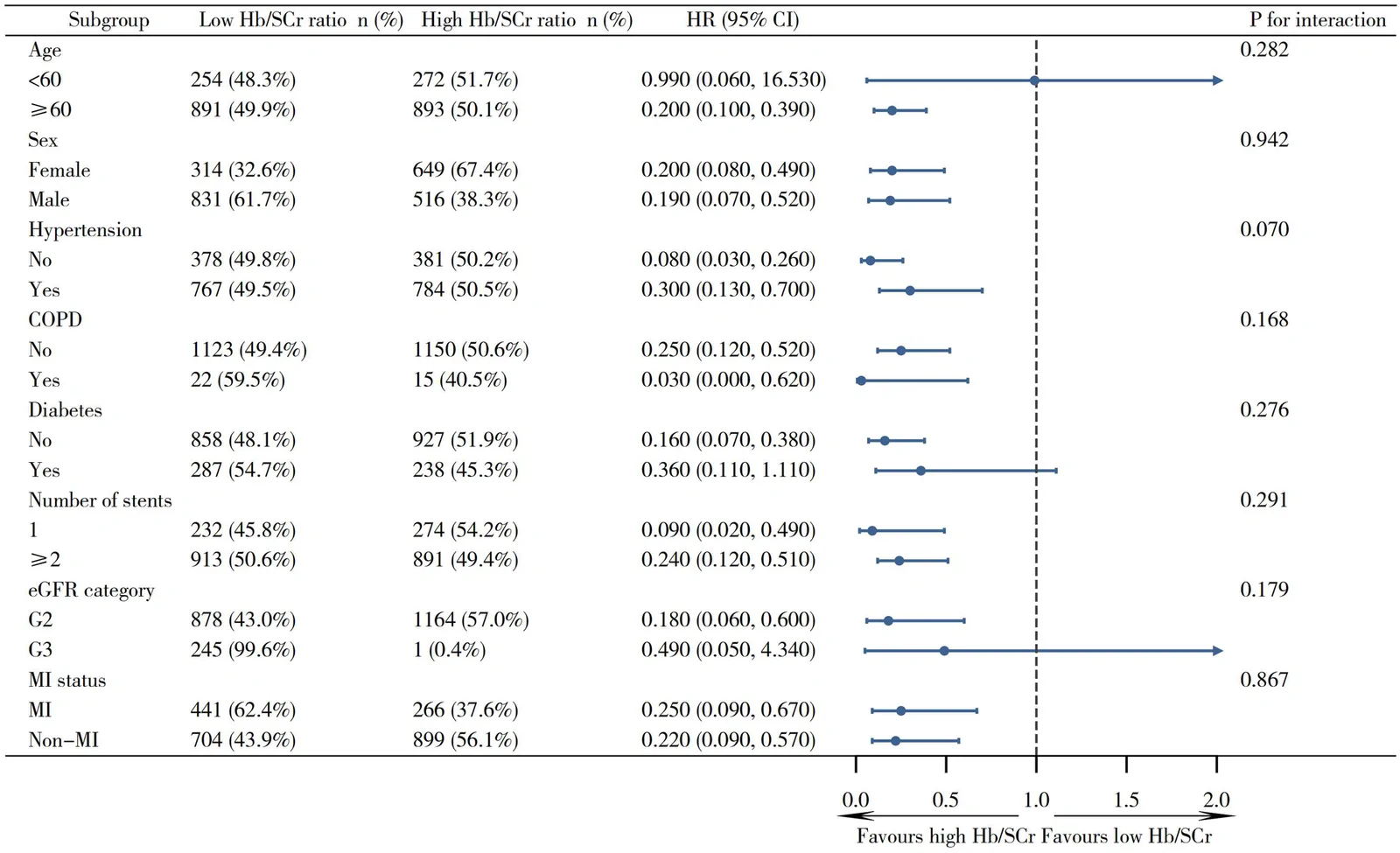
Subgroup analyses of the association between the Hb/SCr ratio and cardiac-cause mortality. Hazard ratios compare the high Hb/SCr group (≥1.60) with the low Hb/SCr group (<1.60). The dashed vertical line represents an HR of 1.0. Estimates for subgroups with sparse patients or events should be interpreted cautiously.

Subgroup analyses demonstrated that, among evaluable strata, the point estimates generally favored the high Hb/SCr group across subgroups defined by age, sex, hypertension, COPD, diabetes, number of stents, MI status, and eGFR category (Figure 5). No statistically significant interactions were observed. Estimates for the eGFR subgroup were imprecise because only one patient in the G3 category had an Hb/SCr ratio ≥1.6 and should therefore be interpreted cautiously.

## Discussion

To our knowledge, this is the first study to evaluate the prognostic value of the hemoglobin-to-serum creatinine (Hb/SCr) ratio in patients with coronary artery disease and reduced kidney function undergoing PCI over long-term follow-up. We found that a lower Hb/SCr ratio was independently associated with higher risks of both cardiac-cause mortality (CCM) and all-cause mortality (ACM) over 60 months. These associations remained significant after adjustment for conventional clinical risk factors and were consistent across multiple clinical subgroups. Using the cohort median value of 1.60 as a pragmatic reference threshold, patients with lower Hb/SCr ratios had significantly poorer long-term outcomes.

Previous studies have demonstrated the prognostic significance of the Hb/SCr ratio in patients undergoing PCI, although most were limited to in-hospital or short-term outcomes and focused primarily on acute myocardial infarction. Numasawa et al. showed in a large national registry study that a lower Hb/SCr ratio was independently linked to higher in-hospital mortality and bleeding risk in non-dialysis patients undergoing PCI (18). In patients with ST-segment elevation myocardial infarction, Spadafora et al. demonstrated that lower Hb/SCr ratios were associated with worse 1-year clinical outcomes after propensity score matching (19). Ji et al. also reported that a diminished Hb/SCr ratio was markedly linked to an elevated risk of long-term major adverse cardiovascular events among patients with ST-segment elevation myocardial infarction (13). Our study extends the follow-up period to 60 months, offering a more complete picture of the prognostic trajectory beyond the acute phase, and focuses specifically on patients with reduced kidney function — a high-risk group in which cardiorenal interactions are particularly pronounced. These findings suggest that the Hb/SCr ratio reflects not only the severity of acute illness but also longer-term physiological reserve in this population.

The potential link between a low Hb/SCr ratio and poor prognosis may be attributable to the complex pathophysiological processes underlying cardiorenal interactions (20, 21). Reduced kidney function is often accompanied by electrolyte abnormalities, including hyponatremia, hyperkalemia, and disorders of calcium and phosphorus metabolism, which may aggravate myocardial stress and increase the risks of arrhythmia and sudden death (9, 22, 23). Additionally, impaired renal erythropoietin production may sustain a persistent anemic state, resulting in chronic myocardial hypoxia. Therefore, a reduced Hb/SCr ratio may function as an integrated marker of cardiorenal interaction, reflecting depletion of cardiorenal compensatory reserve and a substantially increased risk of mortality (24–27).

Several features support the clinical relevance of this marker. Both components are obtained routinely before PCI, so the ratio requires no additional testing, cost, or specialized equipment, and can be calculated at the point of care. Unlike isolated measurements of hemoglobin or creatinine, the ratio captures the combined burden of anemia and impaired kidney function, two major contributors to cardiorenal anemia syndrome. Compared with its individual components, the Hb/SCr ratio showed greater discrimination for CCM than hemoglobin alone and for ACM than serum creatinine alone.

Spline analysis provides guidance on how the ratio might be applied in practice. Restricted cubic spline analysis demonstrated that the adjusted mortality risk increased progressively as the Hb/SCr ratio decreased, supporting a continuous inverse association rather than a discrete threshold effect. In practical terms, a low Hb/SCr ratio may serve as a simple flag prompting closer attention rather than a rule for a specific intervention. Patients in the lowest range could reasonably be considered for more frequent post-discharge follow-up, systematic evaluation of the two contributing abnormalities — including investigation of reversible causes such as iron deficiency, occult bleeding, or nephrotoxic medication use — and careful attention to guideline-directed medical therapy. Conversely, the high negative predictive values observed (98.5% for cardiac-cause and 97.4% for all-cause mortality) suggest that patients with a ratio above 1.6 have a low absolute risk of five-year mortality, although these values should be interpreted in the context of the low event prevalence in this cohort. Because the underlying relationship is continuous rather than threshold-dependent, the ratio is best used to inform a gradient of clinical concern rather than as a binary decision rule.

Several limitations warrant acknowledgment. First, our inclusion criterion (eGFR <90 mL/min/1.73 m^2^) produced a cohort composed predominantly of patients in KDIGO category G2 (88.4%), with fewer in G3 (10.6%) or G4–G5 (1.0%). KDIGO G2 alone does not fulfil the diagnostic criteria for chronic kidney disease in the absence of markers of kidney damage. Kidney function was assessed using a single eGFR measurement obtained at the time of PCI, without confirmation of chronicity or ascertainment of albuminuria; our findings should therefore be interpreted as applying to patients stratified by eGFR rather than to those with confirmed chronic kidney disease. Because most participants were in category G2, generalizability to more advanced kidney disease is limited. In addition, eGFR was estimated using the MDRD equation rather than the CKD-EPI equation currently recommended by KDIGO, which may have influenced category assignment near the boundaries.

Second, this was a retrospective single-center study, and selection bias cannot be excluded; external validity requires confirmation in more diverse populations. Third, the number of outcome events was modest (56 cardiac and 87 all-cause deaths). Although Model 3 included only seven adjustment covariates in addition to the Hb/SCr term, the event-to-parameter ratios were approximately 7.0 for CCM and 10.9 for ACM, and some model instability cannot be excluded. Bootstrap assessment supported the stability of the AUC estimates; however, this analysis did not independently validate the threshold. External validation is therefore required because the reference threshold was selected and evaluated within the same cohort.

Fourth, despite adjustment for multiple clinically relevant variables, residual confounding from unmeasured factors remains possible. In particular, the database did not contain information on discharge medications or longitudinal pharmacotherapy. Guideline-directed medical therapy — including dual antiplatelet therapy, statins, beta-blockers, and renin-angiotensin-aldosterone system inhibitors — substantially influences long-term outcomes after PCI, and differential use or adherence across Hb/SCr groups could have confounded the observed associations in either direction. Anemia-directed therapy and nephroprotective strategies were likewise not captured, despite their direct relevance to the two components of the ratio. Data on anemia subtypes and inflammatory biomarkers were also unavailable, which would have provided additional mechanistic insight.

Finally, the discriminative ability of the Hb/SCr ratio was modest (AUC 0.61–0.66), and the incremental improvement in prediction beyond established clinical variables, while statistically significant for integrated discrimination, was small in absolute terms; continuous net reclassification improvement did not reach significance. Notably, NRI and IDI estimates have limited stability when the number of events is modest. The ratio should therefore be regarded as a complementary marker rather than a standalone predictor. Given the observational design, causality cannot be established. Larger multicenter prospective studies are needed to validate these findings and to determine whether incorporating the Hb/SCr ratio into existing risk scores yields clinically meaningful improvement.

## Conclusion

In patients with coronary artery disease and reduced kidney function undergoing PCI, a lower Hb/SCr ratio was independently associated with higher 5-year cardiac-cause and all-cause mortality, with a continuous dose-response relationship. Because both components are obtained routinely, the ratio can be calculated at no additional cost and may complement existing risk assessment by flagging patients who warrant closer surveillance and systematic evaluation of reversible causes of anemia and kidney dysfunction. Given its modest discriminative ability, it should be regarded as an adjunct rather than a standalone predictor. Prospective multicenter studies are needed to validate the proposed threshold and to determine whether Hb/SCr-guided follow-up strategies improve outcomes.

## Data Availability

The de-identified data supporting the conclusions of this article will be made available by the authors upon reasonable request, without compromising patient privacy and confidentiality.
