# Supplementary material for: Prognostic value of hemoglobin-to-serum creatinine ratio in patients with coronary artery disease and reduced kidney function following percutaneous coronary intervention
Source: Front Cardiovasc Med. 2026 Sep 10;13:1868952. doi: 10.3389/fcvm.2026.1868952 (PMC13600805; doi:10.3389/fcvm.2026.1868952)
Supplement: Supplementary file 1 [file Datasheet1.pdf]

Supplementary Material

Supplementary Table S1. Comparison of discriminative ability between the Hb/SCr ratio and its individual components

Outcome	Marker	AUC (95% CI)	P vs Hb/SCr ratio
Cardiac-cause mortality	Hb/SCr ratio	0.663 (0.585–0.742)	—
	Hemoglobin	0.575 (0.493–0.657)	0.024
	Serum creatinine	0.623 (0.548–0.698)	0.162
All-cause mortality	Hb/SCr ratio	0.614 (0.548–0.680)	—
	Hemoglobin	0.576 (0.512–0.640)	0.284
	Serum creatinine	0.568 (0.503–0.634)	0.037

Pairwise comparisons were performed using DeLong's test. Serum creatinine values were inverted so that all markers shared a common direction of association with the outcomes; this transformation does not alter the area under the curve.

Abbreviations: AUC, area under the receiver operating characteristic curve; CI, confidence interval; Hb/SCr, hemoglobin-to-serum creatinine ratio.

Supplementary Table S2. Bootstrap assessment of the stability of AUC estimates

Outcome	AUC	DeLong 95% CI	Bootstrap 95% CI
Cardiac-cause mortality	0.663	0.585–0.742	0.582–0.741
All-cause mortality	0.614	0.548–0.680	0.549–0.682

Bootstrap resampling was performed with 1,000 replicates. The close agreement between bootstrap-derived and analytically derived confidence intervals indicates stable discriminative performance.

Abbreviations: AUC, area under the receiver operating characteristic curve; CI, confidence interval.

Supplementary Table S3. Assessment of the proportional hazards assumption using scaled Schoenfeld residuals.

Covariate	CCM, categorical	CCM, continuous	ACM, categorical	ACM, continuous
Hb/SCr ratio	0.874	0.273	0.887	0.219
Age	0.262	0.268	0.098	0.101
Sex	0.952	0.959	0.958	0.967
Smoking	0.969	0.976	0.711	0.704
Diabetes	0.305	0.310	0.807	0.812
History of stroke	0.885	0.879	0.605	0.600
LVEF	0.339	0.336	0.260	0.264
Hypertension	0.049	0.048	0.079	0.077
**GLOBAL**	**0.556**	**0.307**	**0.462**	**0.233**

Global tests provided no evidence of a global violation of the proportional hazards assumption, and the Hb/SCr term satisfied the assumption in all models ($P \geq 0.219$). However, hypertension showed a possible covariate-specific departure in the CCM models ($P = 0.049$ and 0.048); this finding should therefore be interpreted cautiously.

Abbreviations: CCM, cardiac-cause mortality; ACM, all-cause mortality; Hb/SCr, hemoglobin-to-serum creatinine ratio; LVEF, left ventricular ejection fraction.

Supplementary Table S4. Association between Hb/SCr ratio quartiles and mortality

Quartile	Hb/SCr range	n	CCM, adjusted HR (95% CI)	P	ACM, adjusted HR (95% CI)	P
Q1 (lowest)	0.13 to 1.40	578	1.00 (reference)	—	1.00 (reference)	—
Q2	>1.40 to 1.60	580	0.46 (0.23–0.93)	0.030	0.62 (0.36–1.07)	0.087
Q3	>1.60 to 1.80	574	0.40 (0.19–0.86)	0.019	0.40 (0.21–0.78)	0.007
Q4 (highest)	>1.80 to 2.56	578	0.34 (0.15–0.79)	0.013	0.60 (0.32–1.12)	0.108
**P for trend**			**0.004**		**0.026**	

Hazard ratios were adjusted for age, sex, smoking, diabetes, hypertension, history of stroke, and left ventricular ejection fraction. Quartiles were defined using left-open, right-closed intervals. P for trend was derived by modelling the quartile rank as a continuous variable. The median Hb/SCr ratio of the cohort was 1.60, corresponding to the boundary between the second and third quartiles.

Abbreviations: CCM, cardiac-cause mortality; ACM, all-cause mortality; HR, hazard ratio; CI, confidence interval.

Supplementary Table S5. Incremental predictive value of the Hb/SCr ratio at 5 years

Metric	CCM, estimate (95% CI)	P	ACM, estimate (95% CI)	P
IDI	0.007 (0.0001–0.0289)	0.048	0.005 (0.0001–0.0217)	0.032
Continuous NRI	0.141 (−0.031 to 0.279)	0.100	0.085 (−0.073 to 0.187)	0.299
Median improvement in risk score	0.002 (−0.001 to 0.011)	0.096	0.001 (−0.002 to 0.006)	0.403

Estimates compare a clinical model comprising age, sex, smoking, diabetes, hypertension, history of stroke, and left ventricular ejection fraction with the same model plus the Hb/SCr ratio, evaluated at 5 years using 500 perturbation resamples.

Abbreviations: CCM, cardiac-cause mortality; ACM, all-cause mortality; IDI, integrated discrimination improvement; NRI, net reclassification improvement; CI, confidence interval.
